# Preoperative empagliflozin and ventricular remodeling after coronary artery bypass grafting in type 2 diabetes: a prespecified analysis of the POST-CABGDM randomized trial

**DOI:** 10.1186/s13098-026-02232-x

**Published:** 2026-07-28

**Authors:** Vinícius Oro Popp, Fabio Grunspun Pitta, Giovanna Daneluz de Brito, Júlia Oro Popp, Letícia Neves Solon Carvalho, Leon Pablo Cartaxo Sampaio, Fabiana Hanna Rached, Bruno Mahler Mioto, Paula Mathias Paulino Bolta, Omar Asdrúbal Vilca Mejía, Luís Alberto Oliveira Dallan, Roberto Kalil Filho, Alexandre Antônio Cunha Abizaid, Carlos Vicente Serrano, Eduardo Gomes Lima

**Affiliations:** 1 Instituto do Coracao, Hospital das Clinicas HCFMUSP, Faculdade de Medicina, Universidade de Sao Paulo, Sao Paulo, SP Brasil; 2 Complexo Hospital São Marcelino Champagnat, Curitiba, Curitiba, PR Brasil; 3 Hospital Universitário Cajuru, Curitiba, PR Brasil; 4 Pontifícia Universidade Católica do Paraná (PUCPR), Curitiba, PR Brasil; 5 Hospital Nove de Julho, Sao Paulo, Brasil

**Keywords:** Type 2 diabetes mellitus, Coronary artery disease, Coronary artery bypass graft, Cardiopulmonary bypass, Sodium-glucose transporter 2 inhibitors, Empagliflozin, Ventricular remodeling, Echocardiography, Left ventricular hypertrophy, Myocardial ischemia-reperfusion injury

## Abstract

**Background:**

Type 2 diabetes mellitus (T2DM) and coronary artery disease (CAD) are associated with adverse ventricular remodeling. Coronary artery bypass grafting (CABG) with cardiopulmonary bypass (CPB) may further contribute to myocardial injury through inflammatory and ischemia–reperfusion mechanisms. Sodium–glucose cotransporter 2 inhibitors (SGLT2i) have demonstrated cardioprotective effects, including reduction in left ventricular mass, but their impact in the perioperative setting of cardiac surgery remains unclear.

**Methods:**

This study is a prespecified secondary analysis of the open-label randomized POST-CABGDM trial (NCT04523064). Patients with T2DM and multivessel CAD undergoing CABG with CPB were randomized to receive empagliflozin 25 mg once daily or standard antidiabetic therapy. SGLT2i were discontinued 3 days before surgery and were not protocol-mandated postoperatively. Transthoracic echocardiography was performed preoperatively and at 1–6 months postoperatively. The primary endpoint was the change in left ventricular mass index (ΔLVMI). Secondary endpoints included changes in left ventricular ejection fraction (ΔLVEF) and ventricular dimensions.

**Results:**

A total of 118 patients were included (53 in the intervention group and 65 in the control group). Baseline characteristics were well balanced. No significant difference was observed in ΔLVMI between groups (− 1.39 ± 18.7 vs. −4.55 ± 16.8 g/m²; *p* = 0.337), and this finding remained unchanged after adjustment for baseline LVMI. Similarly, no differences were found in ΔLVEF or ventricular dimensions. Surgical characteristics and postoperative complications were comparable. In a separate exploratory, non-randomized multivariable analysis, continued use of SGLT2i during follow-up was associated with a higher likelihood of LVMI reduction (OR 4.69; 95% CI 1.20–18.40; *p* = 0.03).

**Conclusions:**

Preoperative empagliflozin use was not associated with significant short-term changes in LVMI, LVEF, or ventricular dimensions after CABG with CPB. Exploratory, non-randomized analyses suggested an association between continued SGLT2i use during follow-up and LVMI reduction, but these findings should be considered hypothesis-generating and require confirmation in prospective studies designed to evaluate sustained perioperative and postoperative therapy.

**Trial registration:**

ClinicalTrials.gov NCT04523064.

**Graphical abstract:**

**Figure:**
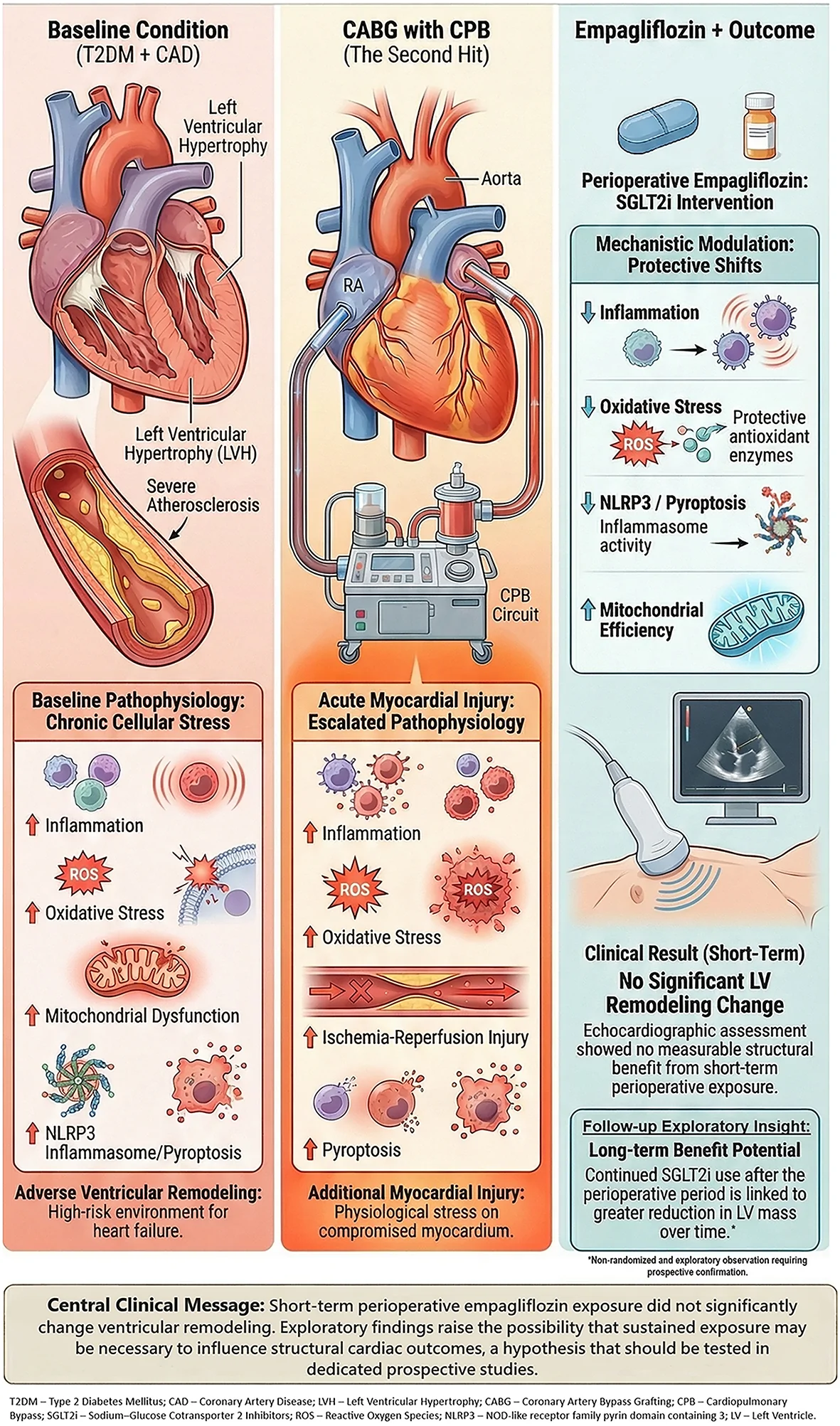

## Background

Coronary artery disease (CAD) and type 2 diabetes mellitus (T2DM) frequently coexist and are associated with worse cardiovascular outcomes, including a higher risk of ischemic events and mortality [1]. In patients with multivessel CAD, coronary artery bypass grafting (CABG) remains a widely used revascularization strategy, particularly in the presence of diabetes [2–6]. Despite its clinical benefits, the combination of underlying disease and surgical intervention, particularly when performed with cardiopulmonary bypass (CPB), is associated with structural and functional myocardial alterations, including inflammation, fibrosis, and adverse ventricular remodeling [7–10]. 

Type 2 diabetes mellitus is associated with myocardial structural changes, including the development of left ventricular hypertrophy, even in the absence of significant hypertension [11]. This finding reflects the underlying mechanisms of diabetic cardiomyopathy, in which chronic inflammation, oxidative stress, and metabolic dysfunction contribute to maladaptive cardiac remodeling [12]. 

In addition to these factors, the use of cardiopulmonary bypass (CPB) during CABG imposes an additional insult to the myocardium, characterized by systemic inflammatory response, oxidative stress, and ischemia–reperfusion injury [13–17]. Together, these mechanisms may contribute to ventricular remodeling in the postoperative period, even in patients without established heart failure [18]. 

Sodium–glucose cotransporter 2 inhibitors (SGLT2i) have emerged as disease-modifying therapies, demonstrating significant reductions in cardiovascular mortality, major adverse cardiovascular events, and hospitalizations for heart failure, as well as robust renoprotective effects by slowing the progression of chronic kidney disease [19–26]. 

Imaging studies, notably the EMPA-HEART CardioLink-6 trial, have shown that empagliflozin promotes significant reductions in indexed left ventricular mass in patients with diabetes and coronary artery disease [27]. Left ventricular hypertrophy is a well-established independent predictor of cardiovascular risk, and its regression is associated with a lower incidence of adverse cardiovascular events [28]. The mechanisms underlying these benefits appear to extend beyond glycemic control, blood pressure reduction, and the hemodynamic effects of natriuresis and osmotic diuresis. Experimental and clinical evidence suggests that SGLT2 inhibitors modulate inflammatory pathways, reduce oxidative stress, improve mitochondrial energy efficiency, and increase nitric oxide bioavailability in the coronary microcirculation [29–31]. Additionally, these agents may influence titin phosphorylation, contributing to improved myocardial relaxation and modulation of pathways related to cardiac hypertrophy [32]. 

Despite these findings, the impact of SGLT2 inhibitors in the perioperative setting of CABG, particularly in patients undergoing CPB, remains poorly explored. Given the environment of intense inflammatory and metabolic activation associated with cardiac surgery, it is plausible that the effects of this drug class may influence ventricular remodeling in this setting.

Therefore, the present study aimed to evaluate the effect of preoperative SGLT2 inhibitor use on echocardiographic parameters of ventricular remodeling in patients with type 2 diabetes undergoing CABG with CPB.

## Methods

### Study design and population

This study is a prespecified secondary analysis of the open-label randomized controlled POST-CABGDM trial (ClinicalTrials.gov: NCT04523064) [33], in which patients were enrolled and randomized between September 2020 and July 2023. The eligible population comprised individuals aged ≥ 18 years with a diagnosis of type 2 diabetes mellitus and multivessel coronary artery disease with a formal indication for coronary artery bypass grafting (CABG) with cardiopulmonary bypass (CPB). Randomization in the parent trial was performed using the REDCap platform, with stratification according to age and baseline renal function, as previously specified in the POST-CABGDM protocol. Treatment allocation was generated electronically after eligibility confirmation.

The main exclusion criteria were estimated glomerular filtration rate < 30 mL/min/1.73 m², need for urgent or emergency surgery, and absence of adequate pre- and/or postoperative echocardiograms for quantitative analysis. For the present analysis, patients in the intervention group whose preoperative echocardiogram was performed more than 28 days after randomization were also excluded, in order to minimize the potential early effects of treatment on baseline measurements. Of the 145 patients enrolled in the POST-CABGDM trial, 27 were excluded from this echocardiographic analysis: 2 because of absence of preoperative transthoracic echocardiography, 10 because the preoperative transthoracic echocardiography in the intervention group had been performed more than 4 weeks after randomization, and 15 because of absence of postoperative transthoracic echocardiography.

The diagnosis of type 2 diabetes mellitus was established according to the American Diabetes Association criteria applicable at the time of the study. Obstructive coronary artery disease was defined as ≥ 70% stenosis in an epicardial coronary artery on coronary angiography, associated with a compatible clinical presentation and/or objective evidence of myocardial ischemia on non-invasive testing, with a formal indication for surgical revascularization.

### Treatment and perioperative management

Participants were randomized to receive empagliflozin 25 mg once daily or standard antidiabetic therapy. Treatment was maintained for at least 3 months prior to surgery, which was performed within 6 months after randomization. SGLT2 inhibitors were discontinued 3 days before surgery in accordance with prescribing information and contemporary safety recommendations for elective surgical procedures [34]. This strategy was prospectively defined in the protocol to minimize the risk of perioperative euglycemic diabetic ketoacidosis in the setting of cardiac surgery with cardiopulmonary bypass, prolonged fasting, and potential hemodynamic and metabolic instability. Protocol-mandated reintroduction in the postoperative period was not performed.

After hospital discharge, diabetes management was left to the discretion of the treating physicians, and the use of SGLT2 inhibitors during follow-up was recorded. Therefore, postoperative SGLT2 inhibitor use was not randomized and was not protocol-mandated. All patients received guideline-directed medical therapy for coronary artery disease. Surgical procedures were performed using CPB, aiming at complete revascularization, according to institutional clinical practice.

### Echocardiographic assessment and outcomes

Transthoracic echocardiography was performed preoperatively, close to the time of randomization, and during follow-up between 1 and 6 months after surgery. Examinations were conducted by experienced echocardiographers in an accredited echocardiography laboratory at a tertiary academic center, within routine clinical practice, in accordance with standardized institutional protocols and the recommendations of the European Association of Cardiovascular Imaging (EACVI) and the American Society of Echocardiography (ASE). Because the parent trial was open-label, formal blinding of echocardiographers to treatment allocation was not implemented for this secondary analysis. The original protocol did not include formal intraobserver or interobserver variability assessments for LVMI, LVEF, or ventricular dimensions.

**Fig. 1:**
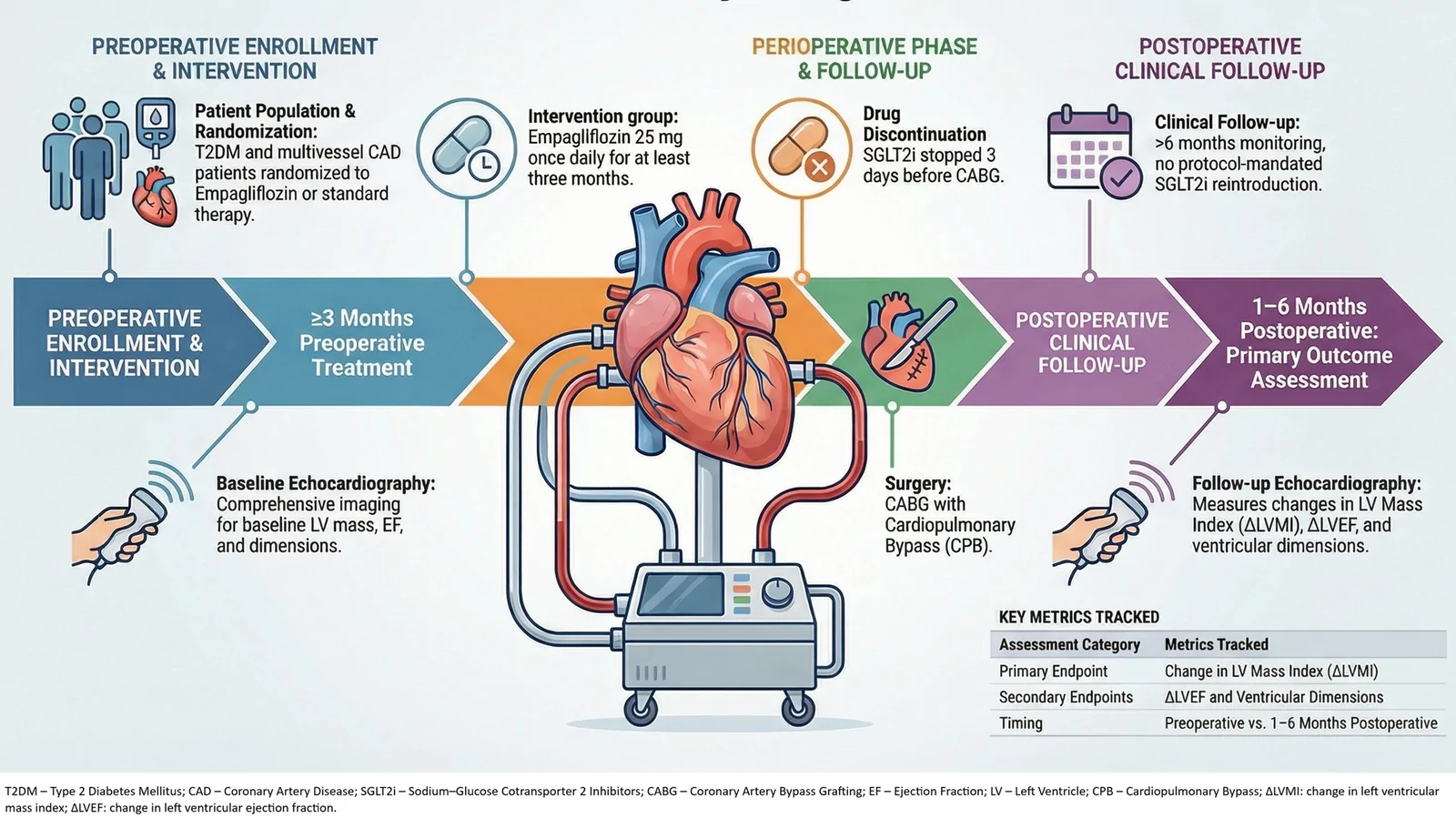
Flow study

The primary endpoint was the change in left ventricular mass index (ΔLVMI), calculated using standardized linear measurements including interventricular septal thickness and posterior wall thickness. Secondary endpoints included changes in left ventricular ejection fraction (ΔLVEF), assessed by the Teichholz method, biplane Simpson method, or visual estimation, as well as changes in left ventricular internal dimension at end-systole (ΔLVIDs) and left ventricular internal dimension at end-diastole. (ΔLVIDd). Clinical outcomes during follow-up, including postoperative complications, were also recorded. The overall study design is illustrated in Fig. 1.

### Statistical analysis

This study represents a pre-specified secondary analysis of a randomized clinical trial. The sample size was determined based on the primary endpoint of the parent study, postoperative acute kidney injury, and was not specifically powered for the present analysis of echocardiographic ventricular remodeling. Because this was a prespecified secondary analysis, an exploratory sample-size calculation was considered using the EMPA-HEART trial as the main available reference evaluating ventricular remodeling associated with empagliflozin [27]. This exploratory calculation used the baseline-to-follow-up correlation assumed in EMPA-HEART (*r* = 0.80) and the LVMI results reported in that trial: mean change in LVMI of − 2.6 ± 7.8 g/m² in the empagliflozin group and − 0.01 ± 5.7 g/m² in the placebo group, with an adjusted between-group difference of − 3.35 g/m² (95% CI − 5.9 to − 0.8; *p* = 0.01). Using these assumptions, a total sample of approximately 100 patients would provide 80% power with a two-sided alpha of 0.05. The correlation between baseline and follow-up LVMI in the present cohort was also assessed using Pearson’s correlation coefficient.

Continuous variables were expressed as mean ± standard deviation or median (interquartile range), as appropriate based on the Shapiro–Wilk test for normality. Comparisons between groups were performed using the Student’s t-test or the Mann–Whitney U test. Categorical variables were compared using the chi-square test.

An exploratory multivariable logistic regression analysis was performed to identify independent predictors of greater reduction in LVMI, defined as the highest quartile of LVMI reduction. This cutoff was not prespecified and was chosen as an exploratory strategy to identify patients with the greatest degree of reverse remodeling without assuming an absolute or relative threshold whose clinical relevance has not been established in this specific population. Variables included in the multivariable model were selected a priori based on clinical relevance as determined by the investigators. Additional sensitivity analyses included ANCOVA with follow-up LVMI as the dependent variable, baseline LVMI as a covariate, and randomized treatment allocation as the factor of interest, as well as testing for interaction between baseline LVMI and treatment group. The interval between surgery and follow-up echocardiography was evaluated both as a continuous variable and as a categorical variable (≤ 90 vs. >90 days). Complementary multivariable linear regression models were also performed using ΔLVMI as a continuous outcome. A two-sided p-value < 0.05 was considered statistically significant. Statistical analyses were performed using SPSS software (version 21.0).

## Results

### Baseline characteristics

#### TTE: Transthoracic echocardiography

**Fig. 2:**
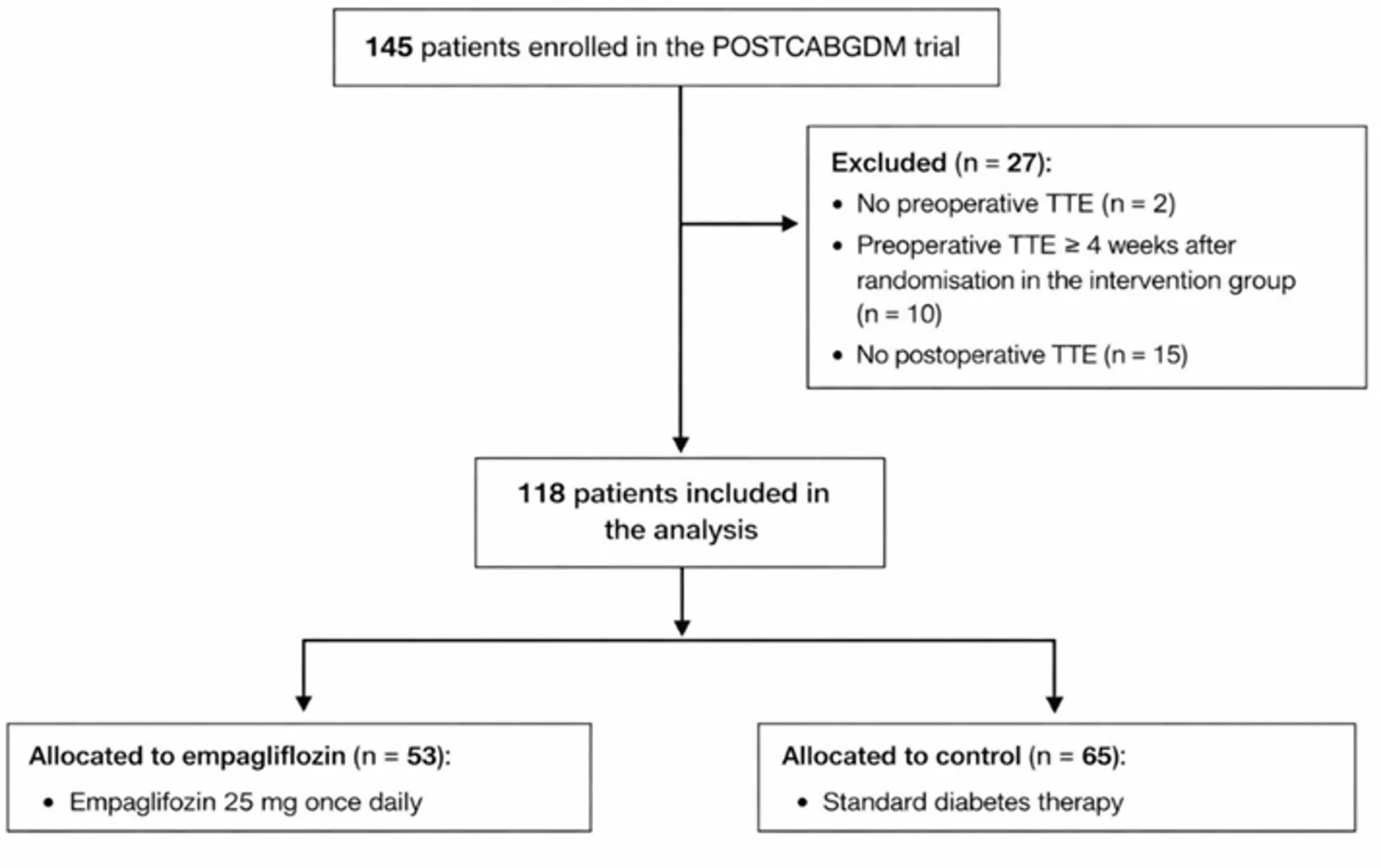
Patient inclusion flowchart

The patient inclusion and randomization flowchart is shown in Fig. 2. The final sample comprised 118 patients, with 53 allocated to the intervention group (SGLT2i) and 65 to the control group. Baseline clinical and demographic characteristics were well balanced between groups (Table 1).

**Table 1:** Baseline demographic, clinical, anatomical, laboratory, and medication characteristics of the study population

Characteristics	Control (*n* = 65)	Empagliflozin (*n* = 53)	*P* valuer de *p*
*Demographics*			
Age (SD)	62 (± 9)	62 (± 8)	0,86
Male sex (%)	41 (63%)	37 (70%)	0,44
Body weight (SD)	82 (± 19)	78 (± 14)	0,22
*Clinical*			
Hypertension (%)	61 (94%)	49 (92%)	0,77
Current or former smoking (%)	38 (58%)	25 (47%)	0,22
Angina status			0,54
CCS class I (%)	10 (15%)	10 (19%)	
CCS class II (%)	31 (48%)	23 (43%)	
CCS class III (%)	12 (18%)	6 (11%)	
No angina (%)	12 (18%)	14 (26%)	
Heart failure status			0,45
NYHA class I (%)	11 (17%)	4 (7%)	
NYHA class II (%)	13 (20%)	14 (26%)	
NYHA class III (%)	3 (5%)	2 (4%)	
No heart failure (%)	38 (58%)	33 (62%)	
Prior stroke (%)	5 (8%)	4 (7%)	0,98
Prior myocardial infarction (%)	35 (54%)	24 (45%)	0,36
Peripheral arterial disease (%)	8 (12%)	6 (11%)	0,87
Diabetes duration > 10 years (%)	36 (55%)	33 (62%)	0,45
Diabetic nephropathy (%)	2 (3%)	0,00	0,20
Diabetic retinopathy (%)	9 (14%)	3 (6%)	0,14
Diabetic neuropathy (%)	18 (28%)	8 (15%)	0,10
*Anatomical*			
Coronary artery disease extent			0,87
Two-vessel disease (%)	8 (12%)	6 (11%)	
Three-vessel disease (%)	57 (88%)	47 (89%)	
*Vital signs at baseline*			
Systolic blood pressure (IQR)	136 (120–150)	136 (120–150)	0,87
Diastolic blood pressure (IQR)	79 (70–84)	80 (70–86)	0,90
Heart rate (IQR)	70 (64–76)	71 (64–77)	0,51
Rate–pressure product (IQR)	9.553 (7.752–10.880)	9.622 (8.640-10.237)	0,67
*Laboratory findings*			
HbA1c at baseline (SD)	7,7 (± 1,4)	7,6 (± 1,5)	0,72
Preoperative HbA1c (SD)	6,8 (± 0,8)	6,5 (± 0,8)	0,14
Serum creatinine (SD)	1,04 (± 0,3)	1,02 (± 0,2)	0,72
Total cholesterol (SD)	156 (± 40)	158 (± 51)	0,80
LDL-C (SD)	84 (± 30)	88 (± 42)	0,54
HDL-C (SD)	40 (± 8)	41 (± 10)	0,42
Triglycerides (IQR)	133 (92–186)	110 (82–188)	0,29
*Medications*			
Insulin (%)	23 (35%)	21 (39%)	0,64
Metformin (%)	58 (89%)	45 (84%)	0,48
Thiazolidinediones (%)	2 (3%)	2 (3,8%)	0,84
Sulfonylureas (%)	34 (52%)	27 (50%)	0,88
DPP-4 inhibitors (%)	12 (18%)	4 (7%)	0,09
GLP-1 receptor agonists (%)	1 (1%)	0,00	0,36
Aspirin (%)	63 (97%)	49 (92%)	0,27
Clopidogrel (%)	12 (18%)	8 (15%)	0,41
High-intensity statin (%)	62 (95%)	52 (98%)	0,42
Beta-blockers (%)	61 (94%)	49 (92%)	0,77
ACE inhibitors (%)	27 (41%)	19 (36%)	0,53
ARBs (%)	32 (49%)	24 (45%)	0,67

SD, standard deviation; CCS, Canadian Cardiovascular Society; NYHA, New York Heart Association; IQR, interquartile range; HbA1c, glycated hemoglobin A1c; LDL-C, low-density lipoprotein cholesterol; HDL-C, high-density lipoprotein cholesterol; DPP-4, dipeptidyl peptidase-4; GLP-1, glucagon-like peptide-1; ACE, angiotensin-converting enzyme; ARB, angiotensin receptor blocker

The mean age was 62 ± 9 years in the control group and 62 ± 8 years in the intervention group (*p* = 0.86), with a predominance of male patients (63% vs. 70%, respectively; *p* = 0.44). The vast majority of patients had three-vessel coronary artery disease (88% in the control group vs. 89% in the intervention group; *p* = 0.87). Most patients did not have a prior clinical diagnosis of heart failure at baseline (58% in the control group vs. 62% in the intervention group; *p* = 0.45).

Clinical and therapeutic profiles were similar between groups, as reflected by comparable glycemic control (preoperative glycated hemoglobin 6.8% ± 0.8 vs. 6.5% ± 0.8; *p* = 0.14) and renal function (serum creatinine 1.04 ± 0.3 mg/dL vs. 1.02 ± 0.2 mg/dL; *p* = 0.72).

### Preoperative echocardiographic characteristics and outcomes

No statistically significant differences were observed between groups in preoperative echocardiographic parameters (Table 2). However, there was a trend toward higher baseline left ventricular mass index (LVMI) in the control group compared with the intervention group (94.23 ± 24.9 g/m² vs. 86.85 ± 19.4 g/m², respectively; *p* = 0.08).

Regarding the primary endpoint, no significant difference was observed in the change in LVMI (ΔLVMI) between groups (intervention: −1.39 ± 18.7 g/m² vs. control: −4.55 ± 16.8 g/m²; *p* = 0.337) (Table 3). Because baseline LVMI was numerically higher in the control group, an additional ANCOVA was performed using follow-up LVMI as the dependent variable, baseline LVMI as a covariate, and randomized treatment allocation as the factor of interest. Baseline LVMI was strongly associated with follow-up LVMI (F = 75.78; *p* < 0.001), whereas empagliflozin allocation was not significantly associated with follow-up LVMI after adjustment for baseline values (F = 0.85; *p* = 0.359). No significant interaction was observed between baseline LVMI and randomized treatment group (F = 0.85; *p* = 0.360). Similarly, no significant differences were found in secondary echocardiographic outcomes, including change in left ventricular ejection fraction (ΔLVEF median: 0 [IQR − 4 to 5] vs. 0 [IQR − 3 to 3]; *p* = 0.959), left ventricular internal dimension at end-systole (ΔLVIDs: −0.32 ± 4.5 mm vs. −1.45 ± 4.5 mm; *p* = 0.179), and left ventricular internal dimension at end-diastole (ΔLVIDd: −0.81 ± 5.0 mm vs. −1.58 ± 4.2 mm; *p* = 0.366).

**Table 2:** Preoperative and follow-up echocardiographic parameters in both randomized groups

Characteristics	Control (*n* = 65)	Empagliflozin (*n* = 53)		*P* valuer de *p*
**Preoperative echocardiogram**				
LA anterooposterior dimension (SD)	40 (± 5)		39 (± 5)	0,17
RV basal diameter (SD)	34 (± 4)		33 (± 3)	0,75
LVIDd (SD)	51 (± 6)		48 (± 5)	0,35
LVIDs (SD)	35 (± 6)		34 (± 5)	0,15
Left ventricular mass index (SD)	94,23 (± 24,9)		86,85 (± 19,4)	0,08
Relative wall thickness (IQR)	0,38 (0,34 − 0,43)		0,38 (0,35 − 0,43)	0,35
LVEF (IQR)	58 (52,5–62)		57 (49,50–61)	0,64
LV EDV index (SD)	62,09 (± 18,4)		59,85 (± 18,7)	0,57
LV ESV index (IIQ)	24,18 (19,62 − 33,02)		23,11 (17,88 − 30,79)	0,54
**Follow-up echocardiogram**				
LA anterooposterior dimension (SD)	41 (± 5)		39 (± 5)	0,21
RV basal diameter (SD)	34 (± 4)		33 (± 4)	0,28
LVIDd (SD)	49 (± 6)		48 (± 5)	0,27
LVIDs (SD)	34 (± 5)		34 (± 5)	0,87
Left ventricular mass index (SD)	89,55 (± 21,3)		85,45 (± 19)	0,28
Relative wall thickness (IQR)	0,38 (0,35 − 0,43)		0,38 (0,32 − 0,43)	0,48
LVEF (IQR)	57 (50,5–62)		57 (50–61,50)	0,67
LV EDV index (SD)	57,35 (± 14,1)		57,28 (± 11,6)	0,11
LV ESV index (IIQ)	21,93 (17,96 − 27,72)		23,07 (18,90 − 31,37)	0,38
**Intervals (days)**				
Echocardiogram 1 to randomization (IQR)	17 (-17-35)		23 (7-105)	0,04
Randomization to surgery (IQR)	87 (82–103)		89 (80–104)	0,99
Surgery to echocardiogram 2 (IQR)	151 (100–177)		147 (87–180)	0,86

LA: Left atrium; SD: Standard deviation; IQR: Interquartile range; RV: Right ventricle LVIDd: left ventricular internal dimension at end-diastole; LVIDs: left ventricular internal dimension at end-systole; LVEF: Left ventricle ejection fraction; LV EDV: Left ventricle end-diastolic volume index; LV ESV: Left ventricle end-systolic volume index

**Table 3:** Echocardiographic outcomes related to ventricular remodeling

Characteristics	Control (*n* = 65)	Empagliflozin (*n* = 53)		*P* valuer de *p*
ΔLVMI (SD)	-4,55 (± 16,8)	-1,39 (± 18,7)	0,337	
ΔLVEF (IQR)	0 (-3 a 3)	0 (-4 a 5)	0,959	
ΔLVIDd (SD)	-1,58 (± 4,2)	-0,81 (± 5,0)	0,366	
ΔLVIDs (SD)	-1,45 (± 4,5)	-0,32 (± 4,5)	0,179	

ΔLVMI: change in left ventricular mass index; SD: Standard deviation; ΔLVEF: change in left ventricular ejection fraction; IQR: ΔLVIDd: change in left ventricular internal dimension at end-diastole; ΔLVIDs: change in left ventricular internal dimension at end-systole

### Surgical, postoperative, and follow-up characteristics

Surgical characteristics and in-hospital complication rates were similar between groups (Table 4). The cohort was predominantly characterized by complete revascularization (98% in the intervention group vs. 100% in the control group; *p* = 0.27), with comparable cardiopulmonary bypass times (88 ± 32 min vs. 90 ± 27 min; *p* = 0.75) and similar distribution of cardioplegia strategies.

**Table 4:** Surgical, postoperative, and in-hospital characteristics

Characteristics	Control (*n* = 65)	Empagliflozin (*n* = 53)	*P* valuer de *p*
**Surgical**			
Number of bypass grafts			0,71
2 grafts (%)	14 (21%)	12 (23%)	
3 grafts (%)	34 (52%)	24 (45%)	
≥4 grafts (%)	17 (26%)	17 (32%)	
Cardiopulmonary bypass time (SD)	90 (± 27)	88 (± 32)	0,75
Complete revascularization (%)	65 (100%)	52 (98%)	0,27
Use of LITA (%)	63 (97%)	53 (100%)	0,12
Cardioplegia strategy			0,17
Del Nido (%)	31 (52%)	19 (38%)	
Intermittent antegrade blood (%)	26 (43%)	28 (56%)	
Intermittent retrograde blood (%)	2 (3%)	0	
Intermittent antegrade crystalloid (%)	1 (2%)	3 (6%)	
**Postoperative outcomes**			
30-day mortality (%)	0	1 (2%)	0,29
Type 5 myocardial infarction (%)	2 (3%)	1 (2%)	0,24
Postoperative atrial fibrillation (%)	7 (11%)	9 (17%)	0,34
Acute kidney injury			0,15
KDIGO stage 1 (%)	21 (32%)	9 (17%)	
KDIGO stage 2 (%)	3 (5%)	2 (4%)	
Requirement for intravenous insulin (%)	38 (58%)	37 (70%)	0,41
Surgical wound infection (%)	10 (15%)	7 (13%)	0,71
Pneumonia (%)	4 (6%)	5 (9%)	0,5
Urinary tract infection (%)	2 (3%)	3 (6%)	0,35
Use of vasopressors in the IPP (%)	9 (14%)	10 (19%)	0,46
Use of inotropes in the IPP (%)	44 (68%)	38 (72%)	0,22
Peak troponin (IQR)	3.918 (2.464–6.165)	3.417 (1.686–7.131)	0,54
Peak C-reactive protein (IQR)	172 (128–211)	159 (128–206)	0,41
**In-hospital outcomes**			
ICU readmission (%)	4 (6%)	1 (2%)	0,25
Length of hospital stay (IQR)	9 (8–14)	8 (7–13)	0,12

SD, standard deviation; LITA, left internal thoracic artery; KDIGO, Kidney Disease: Improving Global Outcomes; IPP, immediate postoperative period; IQR, interquartile range; ICU, intensive care unit

The incidence of early postoperative complications did not differ significantly between groups, including 30-day mortality (2% vs. 0%; *p* = 0.29), atrial fibrillation (17% vs. 11%; *p* = 0.34), stage 1 acute kidney injury (17% vs. 32%; *p* = 0.15), and length of hospital stay (median 8 vs. 9 days; *p* = 0.12).

During outpatient follow-up (Table 5), clinical variables and blood pressure control remained stable. However, a significantly higher proportion of patients in the intervention group remained on SGLT2 inhibitors compared with the control group (23% vs. 6%, respectively; *p* = 0.009), despite the absence of protocol-mandated continuation of the drug after hospital discharge.

**Table 5:** Clinical characteristics during postoperative outpatient follow-up

Characteristics	Control (*n* = 65)	Empagliflozin (*n* = 53)	*P* valuer de *p*
*Risk factor control*			
Systolic blood pressure (IQR)	130 mmHg (120–142)	130 mmHg (120–150)	0,83
Diastolic blood pressure (IQR)	80 mmHg (71–84)	80 mmHg (70–90)	0,87
Heart rate (IQR)	72 bpm (65–81)	77 bpm (68–82)	0,51
Rate–pressure product (IQR)	9676 (8160–11454)	9880 (8580–11550)	0,42
HbA1c at follow-up (IQR)	6,8% (6,1–8,1)	6,6% (5,8 − 7,7)	0,28
*Medications*			
Insulin (%)	20 (30,77%)	21 (39,62%)	0,31
Metformin (%)	54 (83%)	39 (73%)	0,21
Sulfonylureas (%)	25 (38%)	20 (38%)	0,93
DPP-4 inhibitors (%)	5 (8%)	4 (8%)	0,98
Thiazolidinediones (%)	1 (2%)	0	0,36
SGLT2 inhibitors (%)	4 (6%)	12 (23%)	0,009
Beta-blockers (%)	49 (75%)	45 (85%)	0,2
High-intensity statin (%)	62 (95%)	52 (98%)	0,41
ACE inhibitors (%)	39 (60%)	29 (55%)	0,56
ARBs (%)	12 (18%)	12 (23%)	0,57
Calcium channel blockers (%)	36 (55%)	19 (36%)	0,034
Thiazide diuretics (%)	15 (23%)	5 (9%)	0,049
MRA (%)	7 (11%)	9 (17%)	0,33
Hydralazine (%)	1 (2%)	1 (2%)	0,88
Doxazosin (%)	0	1 (2%)	0,27
Clonidine (%)	1 (2%)	0	0,36
Number of antihypertensive drug classes (SD)	2,46 (± 1)	2,28 (± 1)	0,34

IQR, interquartile range; HbA1c, glycated hemoglobin A1c; DPP-4, dipeptidyl peptidase-4; SGLT2, sodium-glucose cotransporter 2; ACE, angiotensin-converting enzyme; ARB, angiotensin receptor blocker; MRA, mineralocorticoid receptor antagonist; SD, standard deviation

### Multivariable analysis

In the exploratory multivariable logistic regression analysis assessing predictors of greater structural reduction in LVMI, defined as the highest quartile of LVMI reduction (Table 6), baseline clinical variables (including age, preoperative left ventricular ejection fraction, and glycated hemoglobin) and surgical characteristics (including cardiopulmonary bypass time, number of grafts, and type of cardioplegia) were not significantly associated with the outcome. The interval between surgery and follow-up echocardiography was not associated with greater LVMI reduction when assessed as a continuous variable (OR 1.00; 95% CI 0.99–1.01; *p* = 0.95).

**Table 6:** Multivariable analysis of independent predictors of LVMI reduction

Variables	OR	Multivariable		X`OR	Multivariable analysis	
		Initial model IC 95%	p		Final model IC 95%	p
Empagliflozin	0,36	0,11 − 1,18	0,09	0,41	0,14 − 1,19	0,10
Age	1,01	0,93 − 1,09	0,85	-	-	-
HbA1c at baseline	1,08	0,70 − 1,67	0,72	-	-	-
Diabetes duration > 10 years	1,29	0,37 − 4,52	0,69	-	-	-
Serum creatinine at baseline	2,19	0,36 − 13,13	0,39	-	-	-
Preoperative LVEF	0,96	0,90 − 1,02	0,21	0,96	0,91 − 1,01	0,10
Cardiopulmonary bypass time	0,97	0,94 − 1,01	0,11	-	-	-
Number of grafts performed (3)	2,12	0,39 − 11,45	0,38	-	-	-
Number of grafts performed (≥ 4)	3,40	0,45 − 25,53	0,23	-	-	-
Del Nido cardioplegic solution	0,15	0,01–2,40	0,18	-	-	-
Intermittent antegrade blood cardioplegia	0,19	0,01–2,71	0,22	-	-	-
Intermittent retrograde blood cardioplegia	0,86	0,02–44,54	0,94	-	-	-
Peak postoperative troponin	1,00	1,00–1,00	0,27	-	-	-
Peak postoperative C-reactive protein	0,99	0,94 − 1,00	0,14	0,99	0,99 − 1,00	0,13
Surgery to follow-up echocardiogram interval	1,00	0,99 − 1,01	0,95	-	-	-
HbA1c at follow-up	0,87	0,59 − 1,30	0,51	-	-	-
SGLT2i at follow-up	5,68	1,19–26,99	0,03	4,69	1,20 − 18,40	0,03
ACEi at follow-up	0,40	0,12 − 1,33	0,13	0,41	0,15 − 1,14	0,09
Beta-blocker at follow-up	0,69	0,18 − 2,68	0,59	-	-	-

OR, odds ratio; HbA1c, glycated hemoglobin A1c; SGLT2i, sodium-glucose cotransporter 2 inhibitor; LVEF, left ventricular ejection fraction; ACEi, angiotensin-converting enzyme inhibitor

In an additional sensitivity analysis incorporating time to follow-up echocardiography as a categorical variable (≤ 90 vs. >90 days), timing of echocardiographic follow-up was also not independently associated with the outcome (OR 3.68; 95% CI 0.48–28.33; *p* = 0.211) and was not retained in the final backward model. Continued use of SGLT2 inhibitors during follow-up was the only independent predictor associated with a significantly higher likelihood of LVMI reduction (OR 4.69; 95% CI 1.20–18.40; *p* = 0.03).

In complementary multivariable linear regression models using ΔLVMI as a continuous outcome, continued SGLT2 inhibitor use during follow-up showed estimates in the same direction but did not reach statistical significance either in the model adjusted using the same covariates included in the logistic regression analysis (β = −9.97 g/m²; 95% CI − 20.65 to 0.71; *p* = 0.067) or in the reduced model (β = −8.39 g/m²; 95% CI − 17.79 to 1.00; *p* = 0.079).

## Discussion

In this prespecified secondary analysis of patients with type 2 diabetes mellitus and multivessel coronary artery disease undergoing coronary artery bypass grafting with cardiopulmonary bypass, preoperative use of empagliflozin was not associated with significant differences in echocardiographic parameters of ventricular remodeling compared with standard therapy. In a separate exploratory, non-randomized multivariable analysis, continued use of SGLT2 inhibitors during outpatient follow-up was associated with a higher likelihood of left ventricular mass reduction. However, because postoperative SGLT2 inhibitor use was not randomized or protocol-mandated, this observation should be interpreted as hypothesis-generating rather than as evidence of a causal treatment effect.

The absence of a significant effect in the overall analysis can be understood in light of the complex pathophysiological milieu underlying myocardial remodeling in patients with diabetes undergoing surgery with cardiopulmonary bypass. Diabetes is associated with a chronic state of inflammation, oxidative stress, and myocardial metabolic dysfunction, involving pathways such as hyperactivation of the sodium–hydrogen exchanger (NHE-1), intracellular calcium overload, mitochondrial dysfunction, and activation of the NLRP3 inflammasome, ultimately leading to inflammatory cell death through pyroptosis. This baseline condition is further exacerbated by the additional ischemia–reperfusion insult inherent to cardiopulmonary bypass, characterized by intense release of reactive oxygen species and inflammatory mediators, amplifying myocardial injury [7, 14, 35–37]. 

In this context, endothelial dysfunction and reduced nitric oxide bioavailability impair intracellular signaling mediated by soluble guanylate cyclase and protein kinase G, resulting in titin hypophosphorylation and increased myocardial stiffness. These mechanisms directly contribute to diastolic dysfunction, progression of left ventricular hypertrophy, and adverse remodeling observed in this population [38–40]. 

SGLT2 inhibitors exert pleiotropic effects that may interfere with these pathological pathways, including inhibition of NHE-1, improvement of ionic homeostasis and mitochondrial function, and modulation of cardiac energy metabolism through increased utilization of ketone bodies, a more energy-efficient substrate. Experimental evidence also suggests activation of antioxidant pathways, such as the Nrf2 axis, with consequent reduction in oxidative stress and inhibition of NLRP3 inflammasome activation, contributing to attenuation of pyroptosis and restoration of pathways associated with myocardial relaxation [41–44]. 

Despite this strong biological plausibility, our findings suggest that limited exposure restricted to the perioperative period may be insufficient to translate these effects into measurable structural changes in the short term. This concept is supported by studies in acute myocardial injury settings, such as the EMPACT-MI and DAPA-MI trials, in which initiation of SGLT2 inhibitors after myocardial infarction was not associated with clear early clinical benefit, highlighting the importance of treatment duration and pathophysiological context in the manifestation of the class effects [45, 46]. 

In the present trial, mandatory discontinuation of SGLT2 inhibitors 3 days before surgery was prospectively defined as a safety strategy to reduce the risk of perioperative euglycemic diabetic ketoacidosis, particularly in the context of prolonged fasting, cardiopulmonary bypass, and potential hemodynamic and metabolic instability. Nevertheless, this approach, together with the absence of a protocol-mandated postoperative reintroduction strategy, may have reduced cumulative exposure to empagliflozin during a potentially relevant period for cardiometabolic and cardiac remodeling effects.

Conversely, evidence from imaging studies, such as the EMPA-HEART and DAPA-LVH trials, has demonstrated significant reductions in left ventricular mass with prolonged use of these agents in patients with diabetes [27, 47]. These findings have been corroborated by meta-analyses of randomized trials confirming the role of SGLT2 inhibitors in reversing left ventricular hypertrophy [48]. Notably, these effects appear to be more pronounced in patients with established hypertrophy, suggesting that baseline remodeling severity may influence the magnitude of therapeutic response [28]. 

This observation is particularly relevant to the interpretation of our results, as most patients in our cohort did not exhibit significant left ventricular hypertrophy or heart failure at baseline. In addition, the control group presented numerically higher baseline left ventricular mass, which may have favored greater proportional regression during follow-up. Baseline and follow-up LVMI measurements in the present cohort showed a significant Pearson correlation (*r* = 0.67; 95% CI 0.56–0.76; *p* < 0.001), supporting both the exploratory sample-size rationale based on EMPA-HEART and the relevance of accounting for baseline LVMI in the ANCOVA sensitivity analysis. However, in the ANCOVA sensitivity analysis, randomized treatment allocation remained unrelated to follow-up LVMI after adjustment for baseline LVMI, and no significant interaction was observed between baseline LVMI and treatment group. These findings suggest that the numerical baseline imbalance in LVMI did not materially influence the main conclusions of the study. The rigorous control of clinical variables, including blood pressure and glycemic control, in both groups may also have attenuated differences between treatment strategies.

Another important aspect relates to the imaging modality used. While previous studies employed cardiac magnetic resonance imaging, which is considered the reference standard for structural assessment of the left ventricle and is capable of detecting subtle changes in myocardial tissue composition, including reductions in extracellular volume and interstitial fibrosis, the present study relied on two-dimensional echocardiography [49–51]. 

Although widely available and clinically applicable, echocardiography is less sensitive and less reproducible than cardiac magnetic resonance for serial assessment of left ventricular mass, particularly when subtle structural changes are expected over short follow-up periods [50–52]. Beyond more accurate quantification of left ventricular mass, cardiac magnetic resonance enables tissue characterization, including late gadolinium enhancement, quantification of extracellular volume expansion, and assessment of diffuse myocardial changes consistent with edema or interstitial remodeling. Therefore, although no significant differences were observed in conventional echocardiographic parameters, we cannot exclude the possibility that more subtle structural or tissue-level changes occurred but were not detected by the imaging methodology employed.

Future studies using cardiac magnetic resonance, ideally with longer follow-up and dedicated assessment of fibrosis, extracellular volume, and myocardial remodeling, may help clarify the potential effects of SGLT2 inhibitors in the perioperative setting.

In the multivariable analysis, no baseline clinical, surgical, or methodological variables—including cardiopulmonary bypass time, type of cardioplegia, renal function, glycemic control, or timing of follow-up echocardiography—were significantly associated with the outcome. Continued use of SGLT2 inhibitors during follow-up was associated with greater LVMI reduction in the exploratory logistic regression analysis. However, this exposure was neither randomized nor protocol-mandated and was determined by treating physicians after completion of the study intervention. In addition, continuation of therapy after hospital discharge may have been influenced by clinical status, physician preference, medication access, and economic factors. Accordingly, this analysis is susceptible to confounding by indication and residual confounding. The complementary linear regression models using ΔLVMI as a continuous outcome showed estimates in the same direction, but neither reached statistical significance after multivariable adjustment. These findings reinforce the exploratory and hypothesis-generating nature of the postoperative SGLT2 inhibitor analysis.

This study has important limitations. Although it represents a prespecified secondary analysis of a randomized clinical trial, the study was not originally designed to assess ventricular remodeling outcomes, which limits statistical power and the ability to detect subtle between-group differences. Accordingly, the findings should be interpreted as exploratory and hypothesis-generating. The parent trial was open-label, and formal blinding of echocardiographic assessment was not implemented for this secondary analysis. In addition, the original protocol did not include formal intraobserver or interobserver variability assessments for LVMI, LVEF, or ventricular dimensions. Although all examinations were performed by experienced echocardiographers within standardized institutional practice, echocardiography has lower reproducibility than cardiac magnetic resonance for serial quantification of left ventricular mass, potentially reducing sensitivity to detect subtle structural changes over time [52]. 

The conduct of the study during the COVID-19 pandemic may have affected follow-up logistics and imaging acquisition. The broad window for postoperative echocardiographic assessment may have introduced temporal heterogeneity, although timing of follow-up imaging was not independently associated with LVMI reduction in sensitivity analyses. Furthermore, the rapid evolution of evidence and recommendations regarding SGLT2 inhibitor use over the study period influenced the intervention design. At the time of trial conception, the benefits of this drug class in non-diabetic populations, such as those with heart failure or chronic kidney disease, were not yet well established, in contrast to the current landscape.

In addition, the absence of protocol-mandated reintroduction of the drug in the postoperative period partly reflected limited access, as these agents were not widely available in the Brazilian public healthcare system during the study period. Finally, continued SGLT2 inhibitor use during follow-up was non-randomized and should not be interpreted as evidence of a causal treatment effect.

Despite these limitations, our findings contribute to the understanding of SGLT2 inhibitor use in a clinical setting characterized by intense inflammatory and metabolic stress. The randomized comparison did not demonstrate significant short-term changes in echocardiographic parameters of ventricular remodeling with preoperative empagliflozin. The exploratory association between continued SGLT2 inhibitor use during follow-up and LVMI reduction should be regarded as hypothesis-generating.

Future studies with dedicated prospective designs, systematic perioperative and postoperative treatment strategies, monitoring for metabolic safety events such as euglycemic diabetic ketoacidosis, longer follow-up, and more sensitive imaging modalities are warranted to better define the impact of this therapeutic strategy in the context of cardiac surgery.

## Conclusion

In patients with type 2 diabetes mellitus undergoing coronary artery bypass grafting with cardiopulmonary bypass, preoperative use of empagliflozin was not associated with significant short-term changes in LVMI, LVEF, or ventricular dimensions. The exploratory, non-randomized observation relating follow-up SGLT2 inhibitor use to LVMI reduction should be interpreted only as hypothesis-generating and requires confirmation in dedicated prospective studies.

## Data Availability

The datasets used and/or analyzed during the current study are available from the corresponding author on reasonable request.

## Abbrevations

T2DM: Type 2 diabetes mellitus
CAD: Coronary artery disease
LV: Left ventricular
CPB: Cardiopulmonary bypass
SGLT2i: Sodium–glucose cotransporter 2 inhibitors
CABG: Coronary artery bypass grafting
ΔLVMI: Change in left ventricular mass index
ΔLVEF: Change in left ventricular ejection fraction
ΔLVIDs: Change in left ventricular internal dimension at end-systole
ΔLVIDd: Change in left ventricular internal dimension at end-diastole
